# Applicability of care quality indicators for women with low-risk pregnancies planning hospital birth: a retrospective study of medical records

**DOI:** 10.1038/s41598-020-69346-8

**Published:** 2020-07-27

**Authors:** Kayo Ueda, Toshiyuki Sado, Yoshimitsu Takahashi, Toshiko Igarashi, Takeo Nakayama

**Affiliations:** 1 Department of Health Informatics, Kyoto University School of Public Health, Yoshida Konoe-cho, Sakyo-ku, Kyoto, 606-8501 Japan; 2 Department of Nursing Women’s Health and Midwifery, Faculty of Nursing, Nara Medical University School of Medicine, 840 Shijo-cho, Kashihara, Nara 634-8521 Japan; 3 Department of Obstetrics and Gynecology, School of Medicine, Nara Medical University, 840 Shijo-cho, Kashihara, Nara 634-8521 Japan

**Keywords:** Epidemiology, Health services

## Abstract

Practices for planned birth among women with low-risk pregnancies vary by birth setting, medical professional, and organizational system. Appropriate monitoring is essential for quality improvement. Although sets of quality indicators have been developed, their applicability has not been tested. To improve the quality of childbirth care for low-risk mothers and infants in Japanese hospitals, we developed 35 quality indicators using existing clinical guidelines and quality indicators. We retrospectively analysed data for 347 women in Japan diagnosed with low-risk pregnancy in the second trimester, admitted between April 2015 and March 2016. We obtained scores for 35 quality indicators and evaluated their applicability, i.e., feasibility, improvement potential, and reliability (intra- and inter-rater reliability: kappa score, positive and negative agreement). The range of adherence to each indicator was 0–95.7%. We identified feasibility concerns for six indicators with over 25% missing data. Two indicators with over 90% adherence showed limited potential for improvement. Three indicators had poor kappa scores for intra-rater reliability, with positive/negative agreement scores 0.94/0.33, 0.33/0.95, and 0.00/0.97, respectively. Two indicators had poor kappa scores for inter-rater reliability, with positive/negative agreement scores 0.25/0.92 and 0.68/0.61, respectively. The findings indicated that these 35 care quality indicators for low-risk pregnant women may be applicable to real-world practice, with some caveats.

## Introduction

No serious differences in clinical outcomes such as infant mortality and morbidity have been reported among low-risk pregnant women giving birth at home, in a midwifery unit, or in an obstetrics unit^1–5^. However, childbirth care practices for women with low-risk pregnancy vary according to birth setting, medical professional, and organizational system. Women with low risk who are planning a birth at home or in a midwifery unit are more likely to have a vaginal birth and to receive less unnecessary medical intervention than women with planned births in an obstetrics unit^6^. In addition, women receiving midwife-led continuous care by the same midwife or team of midwives from pregnancy until the early parenting period report greater satisfaction^7^. In all cases, it is critical to refrain from unnecessary interventions, such as caesarean sections and episiotomies^8–10^.

To improve quality of care, quality indicators have been widely used in many clinical fields. A quality indicator is defined as “a measurable element of practice performance for which there is evidence or consensus that it can be used to assess the quality, and hence change in the quality, of care provided”^11,12^. Quality indicators for maternal and perinatal hospital care have been developed mainly for high-risk pregnancy using the consensus method^13–16^.

In Japan, 98% of women give birth in hospitals^17^, where midwife-led continuous care for low-risk woman is monitored by obstetricians. Among midwives, 87% of midwives works at hospital and clinics^18^. Midwives in Japan are not legally allowed to perform interventions such as episiotomy, epidural anaesthesia, oxytocin infusion, and instrumental delivery. If necessary, obstetricians from the same hospital provide emergency care. Additionally, care for low-risk pregnancy and childbirth is not covered by insurance in Japan; thus, there are no healthcare claims issued for these types of care. Clinical practices that are covered by the national insurance system can be administratively monitored using claims data; however, data for these low-risk pregnancies are neither publicly accumulated nor evaluated. Types of care that are not included in a claims database have not been adequately investigated with respect to quality improvement. To improve this situation and make such care more accessible, we focused on the importance of clinical data that are available from medical records, as the best method for quality improvement in each medical facility. Under this background, to assess the quality of childbirth care provided for women with low-risk pregnancy who give birth in a hospital, we developed and updated care quality indicators using existing clinical practice guidelines and quality indicators^19,20^. We aimed to demonstrate the applicability of care quality indicators for planned hospital births among women with low-risk pregnancies in Japan.

## Methods

### Study design

This was a retrospective study of medical records.

### Study setting and participants

The study was conducted in one urban and one suburban hospital in Japan. Both hospitals have a perinatal medical centre. A perinatal medical centre is a key facility that provides perinatal and postnatal care to the surrounding area. The facilities contained units and teams that could treat serious illness in an emergency. One hospital was affiliated with a university; the other was a private general hospital. As both hospitals have a midwifery unit and an obstetric unit in the same ward, low-risk pregnant women can select midwife-led continuous care or obstetrician-led care from pregnancy to afterbirth. Low-risk pregnancy has no widely accepted definition. In our previous articles, we have defined low-risk pregnancy as “a pregnant woman with no particular high-risk factors or complications”^19,20^. For women who plan to give birth in a midwifery unit and have had at least three prenatal check-ups during each trimester, obstetricians diagnose abnormalities in the woman or the infant. If necessary, emergency care is provided by obstetricians in the same hospital. Low-risk pregnancy in this setting is also defined as a pregnant woman with no particular high-risk factors or complications^20^.

Using a retrospective medical records review, we collected data on women admitted for delivery in the participating hospitals during the study period of April 1, 2015 to March 31, 2016. The inclusion criteria were as follows: determined to have a low-risk pregnancy during the second trimester, and selection of planned hospital birth and midwife-led continuous care from pregnancy until the early parenting period in a midwifery unit. The exclusion criteria were as follows: aspects or complications of high-risk pregnancy such as multiple pregnancy and premature birth < 37 weeks’ gestation, elective caesarean section before the onset of labour, no antenatal care, or declined to participate in this study. This study used only past medical record. According to the current Ethical Guidelines for Medical and Health Research Involving Human Subjects in Japan and the Declarations of Helsinki, we made our study to be open by posting information in the participating hospitals. This study, with the procedure of waiving individual consent, was approved by the Ethics Committee of Kyoto University Graduate School Faculty of Medicine (No. R0442), the Ethics Committee of Morinomiya University of Medical Sciences (No. 2015-29), and the Ethics Committee of Nara Medical University (No. 1269).

### Outcome and evaluation

#### Quality indicator scores

The quality indicators used in this study were developed by a multidisciplinary team of healthcare professionals and lay mothers, using the RAND/UCLA appropriateness method in 2012^19^. These indicators are focused on process and outcome indicators. Based on new or updated clinical practice guidelines, the quality indicators were updated using modified Delphi methods in 2016, resulting in 35 quality indicators^20^. The care quality indicators for women with low-risk pregnancies who planned to give birth in a hospital are listed in Table 1.

**Table 1:** List of original 35 care quality indicators.

No	Theme of indicator	Direction for improvement	Excluding criteria of target subject
*Antepartum*			
1	Primipara who has enrolled in a childbirth class about antenatal care and delivery by 36 weeks gestation	Higher	
2	Discussed a birth plan	Higher	
3	Woman receiving antibiotic prophylaxis during childbirth if maternal group B streptococcus infections are identified at 33–37 weeks' gestation	Higher	
*Intrapartum*			
4	Initial assessment of labour risk at admission: (1) measuring foetal heart rate more than 20 min, (2) vaginal examination, (3) frequency of construction, (4) woman’s emotional and psychological needs, (5) a part and level of pain including her desire for pain relief, (6) foetal movement	Higher	Women admitted during first labour
5	Assessment during first stage labour: (1) 8-hourly temperature and blood pressure, (2) half‑hourly frequency of contractions and foetal heart rate, (3) vaginal examination 4-hourly or if there is concern about progress or in response to the woman’s wishes, (4) woman’s emotional and psychological needs, including her desire for pain relief	Higher	Women admitted during second labour
6	Assessment during second stage labour: (1) 1-hourly blood pressure and woman’s heart rate, (2) half-hourly frequency of contractions, (3) half-hourly foetal heart rate, (4) frequency of passing urine, (5) vaginal examination 1-hourly or if there is concern about progress or in response to the woman's wishes, (6) woman’s emotional and psychological needs	Higher	
7	Women planning spontaneous vaginal birth in a midwifery ward, and being able to follow that plan	Higher	
8	Women with a term, singleton infant in vertex position delivered by caesarean section	Lower	
9	Women with a term, singleton infant in vertex position delivered by vaginal delivery	Higher	Labour induction, instrument delivery or Kristeller manoeuvre
10	Women with a term, singleton infant in vertex position delivered by instrument delivery	Lower	
11	Women with a term, singleton infant in vertex position delivered by labour induction	Lower	The methods other than using uterotonics
12	Term infants with Apgar score less than 7 at 5 min after birth	Lower	Intrauterine foetal death before starting labour
13	Living infants with birth injuries	Lower	
14	Respiratory support: Resuscitation for asphyxiated term neonate with low oxygen concentrations and oxygen saturation measured by pulse oximetry immediately after birth	Higher	
15	Infants offered the necessary resuscitation in the first minutes after birth, evaluating their condition in line with the Japanese Neonatal Resuscitation Algorithm	Higher	Infant death
16	Women having early skin-to-skin contact with their babies if they wish, soon after birth in secure surroundings	Higher	Women didn’t desire early skin-to-skin contact. Women or infants didn’t meet the criteria of early skin-to-skin contact care Women or infants stopped early skin-to-skin contact care
17	Women having been encouraged and supported to adopt the most comfortable positions throughout second stage labour	Higher	A case where the safety for a infant cannot be ensured
18	Women with perineal tear and no perineorrhaphy	Higher	Caesarean section
19	Second degree perineal laceration	Lower	Caesarean section
20	Third or fourth degree perineal laceration	Lower	Caesarean section
21	Postpartum haemorrhage more than 500 g within 2 h of birth	Lower	Caesarean section
22	Women receiving uterotonics for the prevention of postpartum haemorrhage during the third stage of labour	Higher	Caesarean section
*Postpartum: 1 week after childbirth*			
23	Infants admission to paediatrics department within a week after birth	Lower	Infants with antenatally congenital anomalies
24	Infants that were fed only breast milk at the time of discharge from the hospital	Higher	Infants admitted to paediatrics department or needed to supply formula with medical evidence
25	Infants given formula supplementation without medical rationale from birth to discharge in term infants, even though the woman intended to breastfeed	Lower	Infants admitted to paediatrics department or needed to supply formula with medical evidence
26	Peer review of severe adverse events with medical staff	Higher	
27	Women having a fall during their hospitalization	Lower	
28	Women having a review of their childbirth experience and support with the midwives and other staff who assisted at the birth	Higher	
29	Women switched to receive care provided primarily by obstetricians from midwifery ward	Lower	
30	Women received cessation counselling intervention (including guidance on smoking cessation) if identified as either a tobacco user or passive smoker	Higher	Women transported to or from the other hospital
31	Infants administered vitamin K three times by one month after birth	Higher	Infants admitted to paediatrics department
32	Infants who had been fed only breast milk at the time of the health examination for children of 1 month of age	Higher	Infants admitted to paediatrics department or needed to supply formula with medical evidence
33	Women or infants readmitted within 30 days of discharge	Lower	Women having mental health disorders during pregnancy. Infant death
34	Women being screened for antenatal or postnatal depression using a validated questionnaire	Higher	Women having mental health disorders before pregnancy
35	Women and infants having complete medical records based on all quality indicator	Higher	Women and infants admitting within 24 h

“Higher” means that the quality of care in the facility is better when there is a high proportion of patients who received the intervention among the group who would benefit from it.

“Lower” means that the quality of care is better when there is a low proportion of patients with negative events among the group who should receive this care.

We calculated individual indicator scores using a dichotomous variable with values of 0 or 1 for each participant and each indicator. We calculated the percentage of adherence for each indicator as following equation:

$$ \frac{Number\; of\;participants \;eligible \;for\; indicator\; and\; receiving\; recommended\; or \;non{\text{-}}recommended\; care}{{Number\; of\; participants\; eligible\; for\; indicator \;excluding \;those\; with \;an\; obvious \;reason\; not \;to\; undergo\; the \;process, as \;defined \;by \;the\; indicator}}\,(\% ) $$

We analysed the data for clinical assessment of each indicator at the participant level.

#### Evaluation criteria for applicability

We conducted a practical test of multifaceted applicability using the three criteria of feasibility, improvement potential, and reliability^21–23^. (1) Feasibility signifies the extent to which the required data are easily available or can be collected without burdening staff. (2) Improvement potential is the sensitivity to detect when medical performance has changed, to discriminate among and within subjects. (3) Reliability relates to how well the measure is defined and how precisely it is specified so that it can be consistently implemented by the same or different data collectors. To assess the reliability of quality indicators in this study, the inter- and intra-rater reproducibility was examined.

1. Feasibility
  An indicator was considered “unfeasible” if > 25% of participants (denominator) for an indicator score could not be included because of missing data^24^.
2. Improvement potential
  An indicator was considered “low opportunity for quality improvement (or low sensitivity to change)” if the indicator score percentage was ≥ 90%^24,25^.
3. Reliability
  To assess the intra-rater and inter-rater reliability, we randomly sampled the medical records of 20 mothers from each of the two hospitals (n = 40). A researcher (KU) explained the procedure to the two raters (MT, NN). After completing several training sessions, they independently measured the quality indicators twice a month. The intra-rater and inter-rater reliability was evaluated by two research assistants (MT, NN) by measuring data from the records of selected 20 mothers from each hospital. In parallel, two midwives working at each hospital evaluated 10 records. We primarily used the kappa coefficient and secondarily used agreement score (positive and negative agreement score^26^) (Supplementary information 1). The kappa coefficient criteria were as follows: < 0.40, poor; 0.40 ≤ κ ≤ 0.60, moderate; 0.60 < κ ≤ 0.80, good; and > 0.80, very good)^27^. We also determined the percentage of positive and negative agreement for each indicator. The median, minimum, and maximum score for both agreements in terms of intra-rater and inter-rater reliability were also calculated.

### Data sources and measurement

We retrospectively identified eligible mothers from the clinical records using medical safety and management reports. One researcher (KU) and seven midwives (four of whom worked at the participating hospitals and three who were research assistants) collected the data. The midwives had more than 3 years’ work experience and had received training in data collection. They manually collected indicator-relevant data for women and infants from the records and entered them into an electronic data capture system (REDCap)^28^. We used the data to evaluate the performance of planned hospital birth care for women with low-risk pregnancy. The research assistants measured intra-rater reliability. Inter-rater reliability data for research assistants and midwives working in the participating hospitals were collected more than 1 month after the initial measurement.

### Sample size

We assumed an indicator adherence of 50% (the largest number of medical records or participants needed for adherence) with a confidence level of 95% and a precision estimate of 7.5% and included 167 participants. Multiple facilities were set up to obtain 167 participants per hospital^29^. We randomly selected sample records to assess the reliability of over 10% of the total participant records^24^.

### Statistical analysis

We defined missing data as data not recorded in the clinical records. We performed statistical analysis using JMP® Pro, version 14.0 (SAS Institute, Cary, North Carolina, USA).

## Results

### Participants

**Figure 1:**
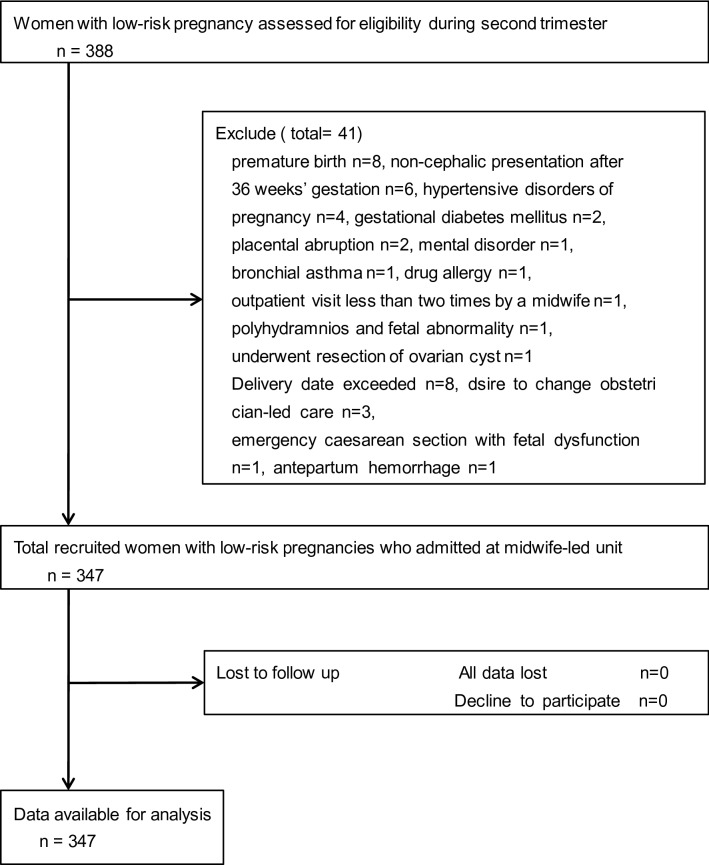
Flow chart for selecting participants.

Of 388 eligible participants, we analysed data for 347 mothers. A flow chart showing participant selection is shown in Fig. 1.

Table 2 shows the characteristics of participating women and infants. The median age for women was 31 years and the median gestational age was 39 weeks. There were 201 multiparous women (58%) and no foetal or neonatal deaths.

**Table 2:** Characteristics of the participating mothers and infants (n = 347).

Characteristics	Median or number (%)	Min–Max
**Mothers**		
Age (years)	31	19–44
Total blood loss (mL)	319	52–1863
Body mass index before pregnancy (kg/m^2^)	19.7	15.3–28.7
Body mass index at delivery (kg/m^2^)	23.9	18.5–31.2
Woman height (cm)	158	147–174
Duration of delivery (hours:minutes)	6:29	1:18–34:15
Pregnancy week when a woman desired to delivery in primary midwifery care	28	18–39
Hospitalization (length of stay) (day)	6	5—16
Nulliparous	146 (42%)	
Multiparous	201 (58%)	
Cigarette use during pregnancy	14 (4%)	
	1 (0%)	
**Infants**		
Birth weight (g)	2,978	2,244–3,968
Birth height (cm)	49	44–53
Gestational age (weeks)	39	37–41
Cord blood arterial acidity (pH)	7.3	7.1–7.5
Base excess (BE) (mmol/L)	− 4.7	− 14.6–5.7
Carbon dioxide tension (PCO_2_) (mmHg)	38.9	14.8–72.7
Oxygen tension (PO_2_) (mmHg)	19.1	9.4–30.2
Infant female	160 (46%)	
Foetal or neonatal death	0 (0%)	

### Quality indicator scores

The scores for each quality indicator are shown in Table 3. The range of adherence to all indicators was 0–95.7%. Of 24 applicable indicators, the highest score (79.5%, 276/347) was found for no. 9 (vaginal delivery). No. 26 (staff peer review of severe adverse events), no. 34 (screening for antenatal or postnatal depression), and no. 35 (having complete medical records based on all quality indicators) had the lowest scores (0%). The mean score for all indicators was 32.6%.

**Table 3:** Scores for the 35 quality indicators: feasibility and improvement potential.

No	Quality indicator	Definition of denominator	Denominator (n)	Missing data (n)	Missing data (%)	Numerator (n)	Adherence (%)
1	Birth class	Primipara	146	23	15.8	104	71.2
2	Birth plan	Pregnant women	347	78	22.5	269	77.5
3	Antibiotic prophylaxis for group B streptococcus infection	Women with group B streptococcus infections at 33–37 weeks' gestation	41	2	4.9	38	92.7^b^
4	Initial assessment of labour risk at admission	Pregnant women admitted for delivery at hospital	347	0	0	332	95.7^b^
5	Assessment during first stage labour	Pregnant women admitted during first labour	342	4	1.2	161	47.1
6	Assessment during first stage labour	Pregnant women admitted for delivery at hospital	347	6	1.7	2	0.6
7	Spontaneous vaginal birth in a midwifery unit	Women planning childbirth at midwifery unit in hospital during second trimester	388	0	0	276	71.1
8	Caesarean section	Pregnant women	347	0	0	7	2.0
9	Spontaneous vaginal delivery	Pregnant women	347	0	0	276	79.5
10	Instrument delivery	Pregnant women	347	0	0	24	6.9
11	Labour induction	Pregnant women	347	0	0	56	16.1
12	Apgar score less than 7 at 5 min after birth	Infants	347	0	0	2	0.6
13	Birth injuries	Living infants	347	11	3.2	10	2.9
14	Respiratory support	Infants with asphyxia after birth	18	5	27.8^a^	1	5.6
15	Japanese Neonatal Resuscitation Algorithm	Infants evaluated to offer the necessary resuscitation in the first minutes after birth	24	7	29.2^a^	17	70.8
16	Early skin-to-skin contact	Pregnant women wished to make early skin-to-skin contact with their babies soon after birth in secure surroundings	347	60	17.3	273	78.7
17	Comfortable positions throughout second stage labour	Women confirmed the safety for baby in comfortable positions	303	63	20.8	226	74.6
18	Perineal tear and no perineorrhaphy	Women had vaginal deliveries	340	0	0	130	38.2
19	Second degree perineal laceration	Woman had vaginal deliveries	340	0	0	72	21.2
20	Third or fourth degree perineal laceration	Woman had vaginal deliveries	340	0	0	4	1.2
21	Postpartum haemorrhage more than 500 g within 2 h of birth	Woman had vaginal deliveries	338	2	0.6	66	19.5
22	Uterotonics for the prevention of postpartum haemorrhage	Woman had vaginal deliveries	340	0	0	131	38.5
23	Admission to paediatrics department within a week after birth	Infants without antenatally congenital anomalies	347	0	0	60	17.3
24	Feeding only breast milk at the time of discharge from the hospital	Infants who didn’t admit to paediatrics department or didn’t need to supply formula with medical evidence	287	0	0	175	61.0
25	Formula supplementation without medical rationale during hospitalization	Infants who didn’t admit to paediatrics department or didn’t need to supply formula with medical evidence	281	0	0	56	19.9
26	Peer review of severe adverse events with medical staff	Women of infant with severe adverse events	10	10	100^a^	0	0.0
27	Women having a fall during their hospitalization	Total number of days while women admitted for birth	2,145	0	0	0	0.0
28	Women having a review of their childbirth experience and support with the midwives and other staff who assisted at the birth	Pregnant women	347	344	99.1^a^	3	0.9
29	Women switched to receive care provided primarily by obstetricians from midwifery ward	Pregnant women	347	0	0	78	22.5
30	Women received cessation counselling intervention	Women who identified a tobacco user or passive smoker and who didn’t transport to or from the other hospital	14	6	42.9^a^	4	28.6
31	Infants administered vitamin K three times by one month after birth	Infants who didn’t admit to paediatrics department	287	282	98.3^a^	4	1.4
32	Feeding only breast milk at the time of the health examination for children of 1 month of age	Infants who didn’t admit to paediatrics department or didn’t need to supply formula with medical evidence	287	0	0	219	76.3
33	Women or infants readmitted within 30 days of discharge	Total number of women and infants hospitalized for birth	794‖	0	0	8	1.2
34	Women being screened for antenatal or postnatal depression using a validated questionnaire	Pregnant women	347	0	0	0	0.0
35	Women and infants having complete medical records based on all quality indicator	Women and infants who didn’t admitting within 24 h	347	0	0	0	0.0

^a^“Unfeasibility” was defined as missing data for > 25% of participants (denominator).

^b^“Low opportunity for quality improvement” was defined as indicator scores ≥ 90%.

### Feasibility

There were six indicators with feasibility concerns: no. 14 (neonatal respiratory support); no. 15 (necessary resuscitation in the first minutes after birth); no. 26 (staff peer review about severe adverse events); no. 28 (having a review of the childbirth experience and support from midwives); no. 30 (mother smokes or receives passive smoking cessation counselling); and no. 31 (administration of vitamin K three times up to 1 month after birth). At the time of data extraction, > 25% of the participants had missing data for these indicators.

### Improvement potential

Two indicators showed a low opportunity for improvement (indicator score ≥ 90%): no. 3 (receiving antibiotic prophylaxis during childbirth if the mother had a group B streptococcal infection) and no. 4 (initial assessment of labour risk on admission).

### Reliability

Table 4 shows the reliability for each quality indicator.

**Table 4:** Results for intra-rater and inter-rater reliability.

No	Intra-rater reliability n = 40			Inter-rater reliability n = 40		
	Kappa	Positive agreement	Negative agreement	Kappa	Positive agreement	Negative agreement
1	1	1	1	0.72	0.91	0.81
2	0.63	0.96	0.67	0.44	0.85	0.57
3	–	1	–	1	1	1
4	0	0	0.96	0.17	0.25	0.92
5	0.68	0.84	0.84	0.29	0.68	0.61
6	0	0	0.97	0	0	0.97
7	1	1	1	1	1	1
8	1	1	1	1	1	1
9	0.86	0.97	0.89	0.79	0.99	0.80
10	1	1	1	1	1	1
11	1	1	1	0.93	0.94	0.98
12	–	–	1	–	–	1
13	1	1	1	0.79	0.80	0.99
14	0	0	0.86	0	0	0.67
15	0	0	0.86	1	1	1
16	0.66	0.92	0.74	0.55	0.88	0.67
17	0.3	0.94	0.33	0.49	0.91	0.57
18	0.79	0.80	0.99	0.88	0.89	0.99
19	0.95	0.97	0.98	1	1	1
20	1	1	1	–	–	1
21	1	1	1	0.93	0.94	0.98
22	0.63	0.77	0.86	0.4	0.68	0.71
23	0.93	0.95	0.98	0.87	0.90	0.97
24	0.9	0.95	0.95	0.85	0.93	0.92
25	0.78	0.82	0.95	0.83	0.88	0.95
26	–	–	1	–	–	1
27	–	–	1	–	–	1
28	1	1	1	–	–	1
29	0.83	0.88	0.95	0.67	0.79	0.88
30	1	1	1	–	–	1
31	0.3	0.33	0.95	0	0	0.95
32	1	1	1	1	1	1
33	− 0.03	0	0.97	1	1	1
34	–	–	1	–	–	1
35	–	–	1	–	–	1

“–” indicates an incalculable positive agreement or negative agreement or kappa score.

Indicators with poor kappa scores (< 0.4) for intra-rater reliability were no. 17 (the most comfortable position during second-stage labour), no. 31, and no. 33 (mother or infant readmitted within 30 days of discharge). Intra-rater reliability kappa scores that were incalculable or 0 were found for ten indicators: no. 3, no. 4, and no. 6 (assessment during second-stage labour), no. 12 (Apgar score less than 7 at 5 min after birth), no. 14, no. 15, no. 26, no. 27 (a fall during hospitalization), no. 34 (screening for antenatal or postnatal depression), and no. 35 (complete medical records based on all quality indicators). Indicators with a poor kappa score (< 0.40) for inter-rater reliability were no. 4 and no. 5 (assessment during first-stage labour). Inter-rater reliability kappa scores that were incalculable or 0 were found for ten indicators: nos. 6, 12, 14, 20, 26, 27, 28, 30, 31, 34, and 35.

The median (range) score for positive agreement intra-rater reliability was 0.95 (0.33–1.00) and for negative agreement intra-rater reliability was 0.99 (0.67–1.00). The lowest positive score (0) was found for the following indicators: no. 4, no. 6, no. 14, no. 15, and no. 33; the second-lowest positive score was 0.33 for no. 31. The lowest negative agreement score (0.33) was for no. 17. The median (range) score for positive agreement inter-rater reliability was 0.91 (0.25–1.00) and for negative agreement inter-rater reliability was 0.98 (0.57–1.00). The lowest positive score (0) was found for no. 6, no. 14, and no. 31. The second-lowest negative score (0.25) was for no. 4. The lowest negative agreement score (0.57) was for no. 2 (birth plan) and no. 17.

Three indicators (no. 17, no 31, and no. 31) with poor intra-rater kappa scores showed positive/negative agreement scores of 0.94/0.33, 0.33/0.95, and 0/0.97, respectively. Two indicators (no. 4 and no 5) with poor inter-rater kappa scores showed positive/negative agreement scores of 0.25/0.92 and 0.68/0.61, respectively.

## Discussion

By extracting the necessary information from 347 existing medical records for mothers and children before assessing quality, we assessed the multifaceted applicability of 35 care quality indicators for planned hospital birth among woman with low-risk pregnancy. The feasibility of 29 indicators was high and 33 indicators showed a high potential for improvement. Although some indicators showed low kappa scores, the high agreement scores indicated that the reliability of these indicators was acceptable. With some caveats, the present practice test supported the applicability of these quality indicators, which were previously developed in Japan.

This is the first study to show the applicability of care quality indicators for planned hospital birth for women with low-risk pregnancy. However, the applicability of these quality indicators for real-world practice needs fully testing before they are disseminated. No studies have tested the applicability of care quality indicators for birth in low-risk women using the consensus method^30–32^. Previous studies that have tested the applicability of quality indicators in general have not fully shared an unified terminology^24,25,33,34^. In the present study, we tested quality indicator applicability in terms of feasibility, potential for improvement, and reliability.

We found that most indicators were feasible as 29 indicators with feasibility had less than 25% of missing data for an indicator score. However, there was concern about the feasibility of the following six indicators owing to the high proportion (> 25%) of missing data: no. 14, no. 15, no. 26, no. 28, no. 30, and no. 31. These indicators showed low feasibility because no data were recorded in the medical charts. If data are prospectively collected with a defined format, there is a lower likelihood of missing or ambiguous data^35,36^. The present practice test revealed that two indicators (no. 3 and no. 4) had a low improvement potential (score of over 90%). This was because these two indicators were practiced almost routinely. We consider this a “ceiling effect”: a phenomenon in which the scores for the quality indicator are near their maximum value and thus impossible to substantially increase. It is also very difficult to evaluate quality improvement or detect differences between the measured scores for such indicators over time and in other hospitals. The present results were based on only two hospitals that actively cooperated with the practice test and may have been conscious about care quality. At present, we cannot be certain that these two indicators showed a ceiling effect and that their measurement was invalid. Accordingly, adherence to the indicators should be examined in a large range of settings to determine whether they should be retained or rejected.

Some of intra-rater and inter-rater reliability showed paradoxical results that low kappa score with high level of agreement^26,37^. Cohen’ kappa is generally used as a method of reproducibility evaluation. The aim of this study is to assess the reliability of quality indicators, that is the inter- and intra-rater reproducibility. Cohen’ kappa is generally used as a method of reproducibility evaluation. However, when the distribution of responses is biased, there are paradoxical cases that kappa score shows low even if the actual proportion of agreement is high, namely. Therefore, we used kappa as primary measure of reliability (the inter- and intra-rater reproducibility), and secondarily used the positive/negative agreement proposed by de Vet et al., considering the possibility of such paradox. Based on both scores of kappa and agreement, quality indicators with low kappa score do not always mean low reliability (reproducibility).

Quality indicator scores were 0 or incalculable kappa scores were close to 0 or 100. All indicators with low kappa scores had positive or negative agreement scores > 0.5 in this study. A low kappa score did not necessarily reflect low reliability for a quality indicator. Therefore, we used positive and negative agreement scores together and assessed reliability considering both scores of them. Agreement scores of 0 were found for no. 4, no. 6, no. 14, no. 15, no. 31, and no. 33, and reflect the very small or large number of participants to which those indicators related. Excluding indicators with an agreement score of 0, indicators with the lowest intra-rater reliability agreement score were no. 31 (positive) and no. 17 (negative). The lowest agreement score for inter-rater reliability was for no. 4 (positive) and for no. 2 and no. 17 (both negative), which may reflect the difficulty of identifying relevant data from the medical records. If clinical staff were given advance notification of surveys of quality indicators and prospective data collection, this may increase adherence to the indicators and onsite data recording, which would improve indicator reliability. An additional reason for low reliability was the composite nature of the indicators (e.g., no. 4). Some indicators comprise two or more individual component measures^23,38^, and so may be characterized by a greater risk of disagreement. However, such composite indicators are meaningful only when all individual components are satisfied; individual itemization would reduce their significance. Therefore, the indicators need to be used as they are, with full knowledge of the risk of low reliability for retrospective record reviews.

We acknowledge several limitations. First, the practice test was conducted in only two hospitals with perinatal medical centres. Both hospitals had enough medical facilities and staff to provide onsite advanced obstetric care for high-risk problems and also midwife-led continuity care. The high level of care in the two participating hospitals may have affected the present findings; data from lower-level hospitals might show more missing data, resulting in lower feasibility according to the criteria. Indicators measured in lower-level hospitals may not show the ≥ 90% or higher adherence found in the present study, and may show greater potential for improvement. Additionally, reliability would be lower for low-quality medical records. Second, the applicability that we examined was limited to feasibility, improvement potential, and reliability. We did not test acceptability and predictive validity (i.e., whether indicators are related to clinical outcomes). However, our multidisciplinary panel evaluated and confirmed validity and acceptability during the development process^19,20^. Adverse maternal or perinatal outcomes for women with low-risk pregnancy are rare, so predictive validity is difficult to establish. Third, although the set of indicators was systematically developed based on existing international practice guidelines and quality indicators, it was only tested in Japan and may not be directly applicable to other countries in its present form. The process used in this study may be useful to test applicability in other settings^39^.

To conclude, the present study showed that the 35 quality indicators for low-risk women planning hospital birth could, with some caveats, be applicable to real-world clinical practice.

## Supplementary information

Supplementary file1 (DOCX 19 kb)

## Data Availability

No sharing data are available.
