# Supplementary material for: Applicability of care quality indicators for women with low-risk pregnancies planning hospital birth: a retrospective study of medical records
Source: Sci Rep. 2020 Jul 27;10:12484. doi: 10.1038/s41598-020-69346-8 (PMC7385256; doi:10.1038/s41598-020-69346-8)
Supplement: Supplementary file 1 — Supplementary file1 (DOCX 19 kb) [file 41598_2020_69346_MOESM1_ESM.docx]

**Supplementary Appendix**

**Applicability of care quality indicators for women with low-risk pregnancies planning hospital birth: a retrospective study of medical records**

Kayo Ueda, MW, MPH, Toshiyuki Sado, MD, PhD, Yoshimitsu Takahashi, MPH, PhD, Toshiko Igarashi, MW, PhD, Takeo Nakayama MD, PhD

Calculation of agreement and kappa scores

Supplementary Table S1

| Rater A | Rater B | | |
| --- | --- | --- | --- |
|  | Positive | Negative | Total |
| Positive | *a* | *b* | *a+b* |
| Negative | *c* | *d* | *c+d* |
| Total | *a+c* | *b+d* | *N* |

- The formulae for calculating positive and negative agreement was as follows:

positive agreement = 2a/(2a + b + c); negative agreement = 2d/(2d + b + c).

The inclusion of cells b and c accounts for the fact that these numbers might be different, so their mean value was taken.

- The formulae for calculating kappa was as follows:

P_0_ = (a + d)/N, P_e_ = {(a + b)(a + c)/N + (c + d)(b + d)/N}/N

Kappa = (P_0_−P_e_)/(1−P_e_)
